# Understanding of healthcare professionals towards the roles and competencies of clinical pharmacists in South Africa

**DOI:** 10.1186/s12913-023-09222-z

**Published:** 2023-03-28

**Authors:** L Crafford, RA Kusurkar, E Bronkhorst, AGS Gous, A Wouters

**Affiliations:** 1 Department of Clinical Pharmacy, School of Pharmacy, Sefako Makgatho Health Sciences University, Ga-Rankuwa, South Africa; 2 Research in Education, Amsterdam UMC location Vrije Universiteit Amsterdam, De Boelelaan 1118, Amsterdam, The Netherlands; 3 LEARN! research institute for learning and education, Faculty of Psychology and Education, VU University Amsterdam, Amsterdam, The Netherlands; 4 Amsterdam Public Health, Quality of Care, Amsterdam, The Netherlands

**Keywords:** Clinical pharmacy, Pharmacist, Interprofessional collaboration, Health professions role, Competencies

## Abstract

**Background:**

Incorporating clinical pharmacists in collaborative medical teams results in better patient treatment and health outcomes. In addition, the understanding of other healthcare professionals (HCPs) towards the role of clinical pharmacists can either facilitate or hinder the implementation and expansion of these services. The main distinction between pharmacists and clinical pharmacists lie in their different scope of duties. This study set out to explore other HCPs’ understanding towards the role of the clinical pharmacists in South Africa, and to identify associated factors.

**Methods:**

An exploratory, survey-based, quantitative study was conducted. A survey assessing HCPs’ understanding based on the competencies and role of a clinical pharmacist was distributed to 300 doctors, nurses, pharmacists and clinical pharmacists. An exploratory factor analysis was carried out to determine the construct validity of the measurement. Items were analysed for grouping into subscales through principal components analysis. Differences in the variable scores for gender, age, work experience and previous experience working with a clinical pharmacist were analyzed using independent t-tests. Analysis of variance was used to analyze differences in the variable scores for the different HCPs and the different departments of work in the hospital.

**Results:**

The factor analysis yielded two separate subscales, measuring HCPs’ (n = 188) understanding towards the *role* of a clinical pharmacist, as well as the *competencies* of a clinical pharmacist. Doctors (85, n = 188) (p = 0.004) and nurses (76, n = 188) (p = 0.022), working in both surgical and non-surgical units, had significantly poorer understanding of the role of clinical pharmacists than clinical pharmacists (8, n = 188) and pharmacists (19, n = 188) (p = 0.028). Where specific clinical pharmacist activities were described, 5–16% of pharmacists were unsure whether an activity forms part of a clinical pharmacist’s role. Over 50% of the clinical pharmacists disagreed that their role also includes pharmacist’s activities, like stock procurement and control, pharmacy and administrative work, and hospital pharmacy-medication dispensing activities.

**Conclusion:**

The findings highlighted the possible impact of role expectations and lack of understanding among HCPs. A standard job description with recognition from statutory bodies could promote other HCPs, as well as clinical pharmacists’ understanding of their roles. Findings further suggested the need for interventions like interprofessional education opportunities, staff induction programmes and regular interprofessional meetings to foster acknowledgement of clinical pharmacy services, promoting the acceptance and growth of the profession.

## Introduction

Successful implementation of new clinical pharmacy services is associated with the provision of patient care that optimizes medication therapy [1–3] Although the role of the clinical pharmacist has advanced over the past decade, it is still under-developed in parts of Europe, as well as many middle- to low-income countries [4–7]. The main differences between a clinical and a hospital pharmacist lie in their scope of duties. The specialized functions of clinical pharmacists require of them to regularly interact with other HCPs in the ward setting, assessing patients and the prescribed treatment. In contrast hospital pharmacists spend most of their time in the pharmacy preparing medications for patients [8, 9]. Globally spontaneous and efficient collaborations between clinical pharmacists and other healthcare professionals (HCPs) have been crucial in implementing and sustaining new clinical pharmacy services [3, 5, 10–14]. Incorporating clinical pharmacists in collaborative medical teams results in better treatment and health outcomes for both adult and pediatric patients [1, 15, 16]. Furthermore, the acceptance rate of clinical pharmacist interventions is generally higher when the clinical pharmacists are closely integrated in the multidisciplinary team [1, 17]. Work environments in which collegial support is received motivates clinical pharmacists and improves their wellbeing. This supports the implementation of clinical services, prevents possible adverse events (burnout) and keeps the quality of patient care as high as possible [18]. Knowledge, attitude and perceptions of other HCPs towards the role of the clinical pharmacist are important factors that can either facilitate or hinder the implementation and expansion of clinical pharmacy services [5, 13, 14, 19]. Insufficient understanding among other HCPs about clinical pharmacy services could lead to the misperception that clinical pharmacists do not have the relevant expertise and do not provide benefit to the hospital. This results in a lack of support from HCPs [5, 13]. To date, no research has been done to explore the understanding of other HCPs towards the role of the clinical pharmacists in South Africa.

The role defined for a clinical pharmacist includes the following: direct involvement throughout the medication-use process in order to minimize risks, and reduce mortalities with the increasing improvement of patient outcomes [20, 21]. The scope of practice of clinical pharmacists, outlined by the South African Pharmacy Council (SAPC) in 2014, currently includes clinical functions as well as logistical functions. Some activities included in their role are conducting ward rounds for delivering comprehensive medication management services, counselling patients and collaborating with other HCPs to optimize patient outcomes. Additionally, activities like stock procurement and control, pharmacy and administrative work, as well as hospital pharmacy-medication dispensing activities, are also included in their scope of practice [22]. In countries, like Canada, Sweden, the United Kingdom and the United States, research in primary care settings has highlighted the significance of understanding HCPs’ perceptions and expectations of clinical pharmacy services [3, 5, 10–13]. This is important for developing collaborative relationships [5]. A study looking into the working relationship between ward-based pharmacists and other HCPs found that collaborative working relationships are multifaceted and involve a consideration of the professionals’ understanding of the ward pharmacist’s role and capabilities [23]. Likewise, in Kuwait and India, research has shown that a lack of understanding regarding pharmaceutical care could hinder collaborative practice [19, 24]. In turn this could result in the exclusion of clinical pharmacists and their integral role as part of the healthcare team [19, 24] Optimized medication-related outcomes and decreased healthcare costs, furthermore underscores the need for effective working relationships between pharmacists and other HCPs [14, 24–27].

While in many countries the profession of clinical pharmacy is more common [5, 10–12], clinical pharmacy in South Africa has only experienced significant development in the past decade [9]. South Africa has two sectors for healthcare, the private healthcare sector serving only approximately 15% of the population and the public healthcare sector responsible for the majority [20, 21]. Bronkhorst and colleagues (2021) [28] found that staff-shortages are more pronounced in the public than in private institutional sectors. South Africa’s National Department of Health (NDoH) introduced the National Health Insurance (NHI) and its pilot phase commenced in 2012 [29]. The system requires ample pharmaceutical personnel and the direct involvement of clinical pharmacists throughout the medication-use process to ensure continuity of care [20, 21]. However, as of yet, there are no official clinical pharmacy positions available, because the discipline cannot be registered as a specialization by the SAPC. As a result, in the public sector clinical pharmacists have barely been appointed in allocated posts. In order to bridge the gap, the private sector has created ward-based pharmacist or clinical practice pharmacist posts. Clinical pharmacists across public and private sectors have been reported to perform a variety of functions at ward level [28]. Among individuals not appointed in dedicated clinical pharmacy positions, higher amotivation (lack or absence of motivation) for delivering clinical pharmacy services were found. These pharmacists did not feel supported and related to other HCPs, compared to those in dedicated positions [18]. If allocated more ward-based time, the majority of pharmacists in a study by Bronkhorst et al., felt that they can improve the rational use of medication. They felt that standardised positions with specialist certification, as found in the United States, might enhance practice [28]. Studies from around the world support this belief [30–32].

This study set out to explore the understanding of HCPs towards the role of clinical pharmacists. The research questions were as follows: (1) What is the understanding of different HCPs towards the role of clinical pharmacists? (2) Which factors are related to the understanding of HCPs towards clinical pharmacy? (3) How do clinical pharmacists perceive their own role?

## Method

### Study design and setting

An exploratory, survey-based, quantitative study was conducted. The study took place over a period of six months in 2020 at an academic teaching hospital, forming part of the public healthcare sector, located in the Gauteng Province of South Africa.

### Study sample

Convenience sampling was used to include doctors, nurses and pharmacists from the departments of surgery, intensive care, obstetrics and gynaecology, internal medicine, paediatrics and pharmacy.

The following inclusion criteria for the different health care professionals were used:

- Doctors: currently employed for internship or community service, medical officers, registrars and specialists;
- Nurses: currently employed as either professional, enrolled, staff or registered nurses;
- Pharmacists: all pharmacists with or without a Master’s degree, including those employed for internship or community service.

Pharmacist assistants, auxiliary and assistant nurses were excluded because they rarely interact with pharmacists who have an M. Pharm in Clinical Pharmacy, more especially in all clinical related services in the hospital. For this study’s purpose we decided to include the professions that they most commonly encounter.

### Data collection

A total of 300 surveys were manually distributed to different health care professionals in the respective departments. The doctors and nurses received and filled out the survey during their morning meetings, as most convenient for them. Pharmacists working in the hospital pharmacy received the survey during their weekly meetings, taking into account their schedules. Clinical pharmacists not present in the hospital pharmacy were approached in their respective departments.

### Structure of survey

The survey consisted of two questionnaires. The doctors and nurses’ surveys contained similar questionnaires, designed on the basis of healthcare setting, feasibility and practicality and relevant literature review. We aimed to explore the understanding of the healthcare professionals towards the role of clinical pharmacists. The survey had three sections, denoted A, B, and C. As described in Table 1, section A was designed to collect demographic data, section B and C of each survey were designed based on the competencies and role of a clinical pharmacist as outlined by the SAPC and the American College of Clinical Pharmacists (ACCP) [22, 33] and on literature by Kaboli et al. (2006) [34] and Bronkhorst et al. (2018) [7]. These were tailored to the different groups of HCPs, and all responses indicating the HCP’s level of agreement were made on a 5-point Likert scale.

**Table 1:** Structure of the Surveys

	Doctors’ and Nurses’ Survey	Pharmacists’ and Clinical Pharmacists’ Survey
Section A	• Gender • Age • Years of work experience • Previous experience working with a clinical pharmacist • HCP group • Unit/Department of work	• Gender • Age • Years of work experience • Previous experience working with a clinical pharmacist^a^ • HCP group • Unit/Department of work
Section B	• A multiple choice item assessing knowledge on the definition of a clinical pharmacist (with definitions for a pharmacist, as well as a pharmacologist as alternative options)	• 7 items on activities that comprise the role of a clinical pharmacist*
Section C	• 11 items assessing the understanding of these HCP’s towards the role of clinical pharmacists* • 10 items assessing the understanding of these HCP’s towards the competencies of a clinical pharmacist*	• 11 items assessing the understanding of these HCP’s towards the role of clinical pharmacists* • 10 items assessing the understanding of these HCP’s towards the competencies of a clinical pharmacist*

*Indicate the level of agreement on a 5-point Likert scale, 1 = strongly agree; 5 = strongly disagree

The questionnaires aimed to explore the understanding of other HCPs towards the role and competencies of clinical pharmacists, as well as the understanding of clinical pharmacists regarding their own competencies. The questionnaires were piloted and revised through feedback from doctors, nurses and M. Pharm clinical pharmacy students.

### Data analysis

Descriptive statistics were used to assess demographic data such as gender and years of experience. Data were checked for normality distribution and the assumption of a normal multivariate distribution was met. An exploratory factor analysis was carried out on all item scores in the questionnaire to determine the construct validity of the measurement using this questionnaire, as it was not validated before. These items were analysed for grouping into subscales by using principal components analysis using promax rotation with Kaiser normalization, as the factors were not expected to be completely independent of each other [35]. Cronbach’s alphas for internal consistency were computed for establishing the reliability of the subscales.

We tested for differences in the variable scores for gender, age, work experience and previous experience working with a clinical pharmacist using independent t-tests. Differences in the variable scores for the different healthcare professionals and the different units/departments of work in the hospital were tested using analysis of variance (ANOVA). For analysis we have divided the department specialities into four groups; surgical (surgery and obstetrics and gynaecology), non-surgical (intensive care unit, internal medicine and paediatrics), pharmacy and clinical pharmacy. All analyses were performed using the Statistical Package of Social Sciences (SPSS) 27.0.

### Ethical considerations

Ethical approval for this study was received by the Sefako Makgatho Health Sciences University Research and Ethics Committee (SMUREC/P/232/2020:PG). Participation was voluntary, no incentives were provided for participation and the responses were anonymized. All participants gave their informed consent for participation in the study.

## Results

The response rate was 62.6% (188/300), this included 45.2% (85; n = 188) doctors, 40.4% (76; n = 188) nurses, 10.1% (19; n = 188) pharmacists and 4.3% (8; n = 188) clinical pharmacists. The denominators for each of the HCPs were as follows: 102 doctors, 158 nurses, 32 pharmacists and 8 clinical pharmacists. Assessing doctors’ and nurses’ knowledge on the definition of a clinical pharmacist, this study found sixty-nine (81.2%) doctors and 47 (61.8%) nurses chose the correct definition.

**Fig. 1:**
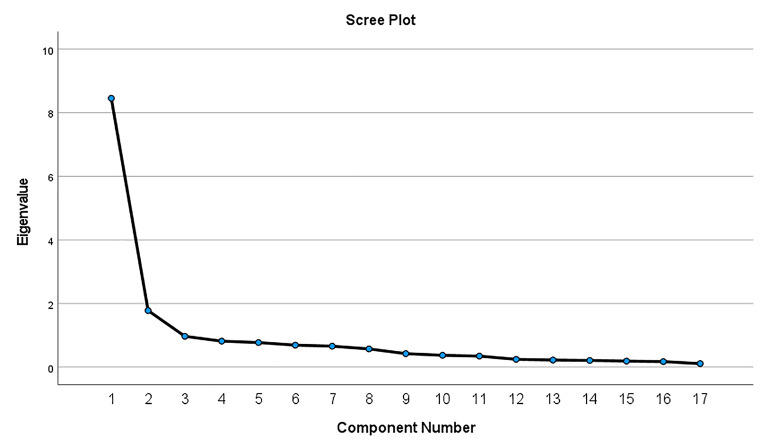
Scree plot of the 17-item survey factor analysis

After re-evaluation of the survey, we decided to delete items 3, 8, 10 and 11, as they did not relate to what we aimed to measure. The Cronbach alpha of the 17-item survey was 0.927. The factor analysis with promax rotation thereafter yielded two separate factors or subscales explaining 49.74% of the variance in the item scores. The criteria for accepting the 2-factor structure were eigenvalues above 1 [36], the scree plot (Fig. 1) and the amount of variance explained by the factors. This resulted in two final subscales.

As shown in Table 2, ten items (i.e. 12, 13, 14, 15, 16, 17, 18, 19, 20 and 21) based on their factor loadings (> 0.40) [35, 36] fitted into subscale 1, measuring HCP’s understanding towards the Role (and attitude) of a Clinical Pharmacist. Whereas, seven items (i.e. 1, 2, 4, 5, 6, 7 and 9) fitted into subscale 2, measuring HCP’s understanding towards the Competencies of a Clinical Pharmacist.

**Table 2:** Factor loadings of the survey items

	Factor 1	Factor 2	
1. Clinical pharmacy is a health science discipline in which pharmacists provide patient care that optimizes medication therapy and promotes health, and disease prevention	0.379	**0.685**	
2. Clinical pharmacy is the priority role of the hospital pharmacists	0.202	**0.664**	
4. Clinical pharmacists are providers of pharmacological therapy information and training to doctors and nurses	0.418	**0.558**	
5. Further formal education or training courses are required for a pharmacist to provide efficient clinical pharmacy services	0.352	**0.514**	
6. Clinical pharmacists carry more responsibility for the patient’s clinical and therapeutic outcomes	0.344	**0.524**	
7. Regular multidisciplinary ward rounds that include a clinical pharmacist create professional relationships and promote collaborative practices	0.354	**0.810**	
9. Doctors and nurses should be encouraged to consult with a clinical pharmacist regarding pharmacotherapy challenges of a patient	0.490	**0.733**	
12. Make use of scientific/clinical evidence as the basis for therapeutic decision-making	**0.824**	0.527	
13. Follow up on and monitor the outcomes of therapeutic plans	**0.835**	0.427	
14. Communicate effectively	**0.845**	0.465	
15. Provide clear and concise consultations to other health professionals	**0.866**	0.455	
16. Communicate with appropriate levels of assertiveness, confidence, empathy, and respect	**0.885**	0.485	
17. Apply knowledge of pharmacoeconomics and risk-benefit analysis to patient-specific and/or population-based care	**0.789**	0.489	
18. Participate in identifying systems-based errors and implementing solutions	**0.876**	0.472	
19. Demonstrate and apply in-depth knowledge of pharmacology, pharmacotherapy, pathophysiology, and the clinical signs, symptoms, and natural history of diseases and/or disorders	**0.881**	0.497	
20. Locate, evaluate, interpret, and assimilate scientific/clinical evidence and other relevant information from the biomedical, clinical, epidemiological, and social-behavioural literature.	**0.865**	0.408	
21. Assess patients, including identifying and prioritizing patient problems and medication-related needs	**0.801**	0.308	

Table 3 shows the population’s demographics with their corresponding mean scores for each subscale.

**Table 3:** Mean scores of healthcare professionals’ understanding regarding the role and attitude of clinical pharmacists, as well as the competencies of clinical pharmacists

Characteristic	No. Respondents, n (%)	Role and attitude of a Clinical Pharmacist Mean (SD)	Competencies of a Clinical Pharmacist Mean (SD)
**Total sample**	188		
**Gender (n = 188)**			
• Female	120 (63.8%)	1.85 (0.799)	1.87 (0.615)
• Male	68 (36.2%)	1.88 (0.694)	2.02 (0.511)
**Age (n = 188)**			
• < 40 years	107 (56.9%)	1.79 (0.689)	1.87 (0.527)
• ≥ 40 years	81 (43.1%)	1.95 (0.844)	2.00 (0.645)
**Years of Work Experience (n = 188)**			
• < 20 years	102 (54.3%)	1.86 (0.680)	1.92 (0.552)
• ≥ 20 years	86 (45.7%)	1.86 (0.852)	1.92 (0.620)
**Previous Experience Working with a Clinical Pharmacist (n = 161)**			
• Yes	61 (37.9%)	1.87 (0.858)	1.76 (0.603)
• No	100 (62.1%)	1.99 (0.711)	2.01 (0.588)
**HCP Group (n = 188)**			
• Doctors	85 (45.2%)	2.01_a_ (0.823)	2.00 (0.568)
• Nurses	76 (40.4%)	1.88_a,c_ (0.705)	1.82 (0.632)
• Pharmacists	19 (10.1%)	1.45_b,c_ (0.487)	1.99 (0.478)
• Clinical Pharmacists	8 (4.3%)	1.09_b_ (0.125)	1.93 (0.374)
**Unit/Department (n = 188)**			
• Surgical	73 (38.8%)	1.91_a_ (0.817)	1.91 (0.632)
• Non-Surgical	88 (46.8)	1.98_a,c_ (0.732)	1.92 (0.583)
• Pharmacy	19 (10.1%)	1.45_a,b_ (0.487)	1.99 (0.478)
• Clinical Pharmacy	8 (4.3%)	1.09_b_ (0.125)	1.93 (0.374)

Mean scores are based on a 5-point Likert scale, on which 1 represented strongly agree and 5 represented strongly disagree.

*p < 0.05.

The means with different subscripts are significantly different from each other, i.e. a mean with subscript “a” is significantly different from a mean with subscript “b” or “c”.

For the demographic variables gender, age and years of work experience all HCPs were included (n = 188), however for the variable “Previous Experience Working with a Clinical Pharmacist” only the doctor and nurse respondents were included, 85.6% (161, n = 188).

Statistically significant differences (P < 0.05) among the different HCPs understanding regarding the role of clinical pharmacists were found, F = 6.130, P < 0.001. Additionally, significant differences were found among the understanding of HCPs working in different units/departments regarding the role of clinical pharmacists, F = 5.754, P < 0.001.

Concerning the different HCPs, we found that doctors’ (P = 0.004) and nurses’ (P = 0.022) understanding towards the role of a clinical pharmacist was significantly poorer to that of the clinical pharmacists. Furthermore, we also found that doctors’ understanding regarding the role of a clinical pharmacist was significantly poorer than that of the pharmacist group (P = 0.016).

Concerning the different units/departments where HCPs worked, we found that doctors and nurses working in the surgical units’ understanding were significantly poorer (P = 0.015) as compared to clinical pharmacists. Additionally, regarding the role of clinical pharmacists, the understanding of doctors and nurses working in non-surgical departments were significantly poorer compared to both clinical pharmacists (P = 0.007) and pharmacists (P = 0.028).

There were no significant differences between any of the independent variables and understanding towards the competencies of clinical pharmacists. Furthermore, no other significant relationships were found, the understanding of HCPs towards the role and attitude and competencies of clinical pharmacists were not associated with gender, age, years of work experience, nor with previous experience working with a clinical pharmacist.

**Fig. 2:**
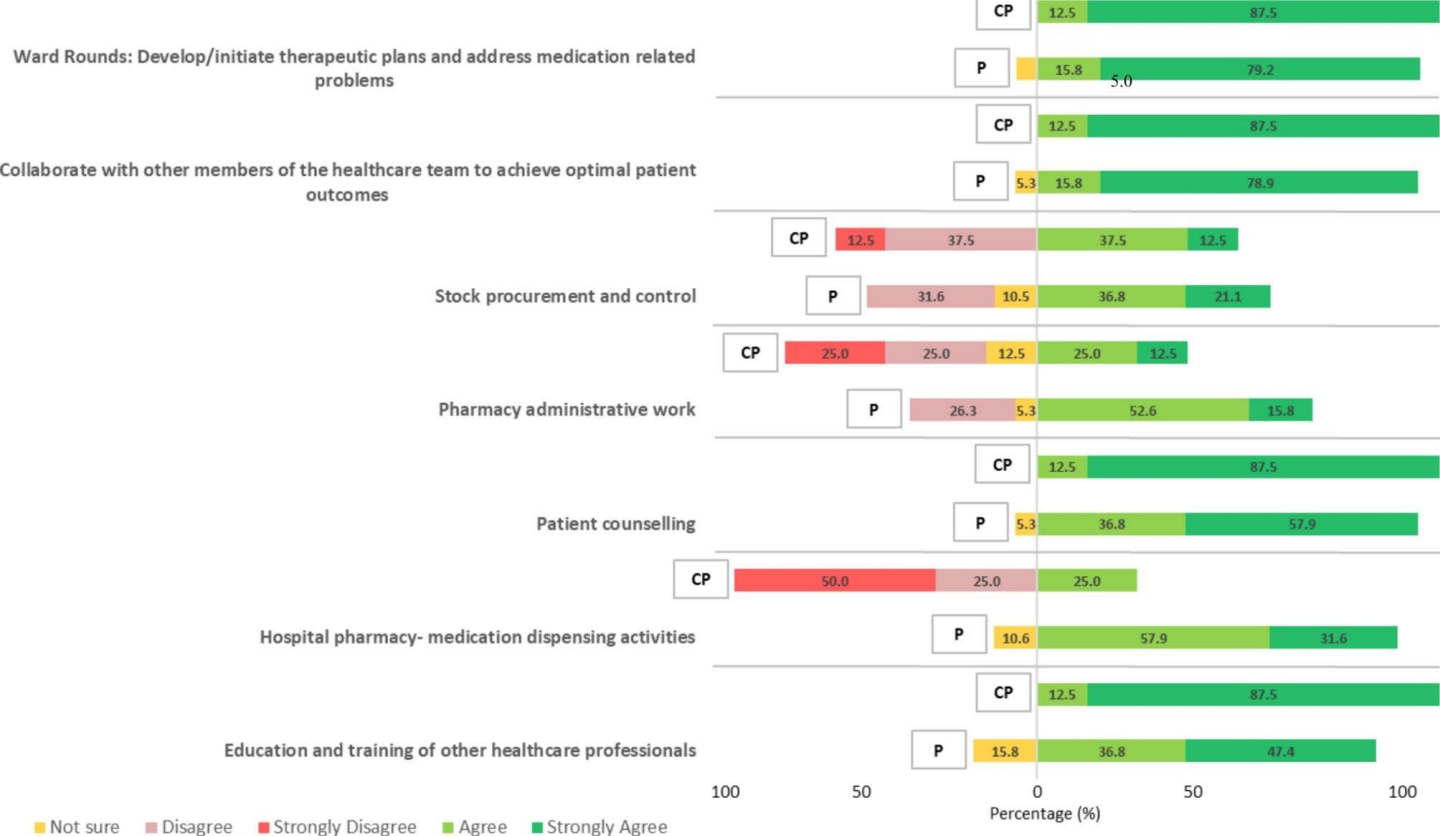
Knowledge of pharmacists and clinical pharmacists regarding activities comprising the role of clinical pharmacist/ Activities comprised in the role of a clinical pharmacist CP = clinical pharmacist; P = pharmacist

The pharmacists’ and clinical pharmacists’ survey included additional questions on specific activities that comprise the role of a clinical pharmacist. As shown in Fig. 2, differences were found between their level of agreement regarding different activities. Concerning every activity described, a percentage of pharmacists were found to be unsure whether the specific activity forms part of the role of a clinical pharmacist. At least half of the clinical pharmacists (50 − 75%) disagreed or strongly disagreed that activities pertaining to the profession of a pharmacist, like stock procurement and control, pharmacy and administrative work, and hospital pharmacy-medication dispensing activities, form part of their role.

## Discussion

The aim of this study was to explore the understanding of HCPs toward the role of clinical pharmacists, identifying the factors that are related to their understanding, as well as the differences and similarities in understanding among different HCPs.

Overall, the mean scores for all HCPs on both subscales were on the positive side. The understanding of the different HCPs towards the role of a clinical pharmacist, the perceptions/understanding of both doctors and nurses’ were poorer compared to that of clinical pharmacists’, and doctors’ understanding compared with that of pharmacists were also significantly poorer. However, in general the mean scores of doctors and nurses were not necessarily poor and no significant differences between the understanding of these two HCPs were found. In South Africa, clinical pharmacy is still in its infancy in public sector hospitals, not only is there an absence of official job descriptions, but the existing human resource shortage and suboptimal or lack of technical support staff makes it challenging to allocate clinical pharmacists to the wards full-time [7, 9, 37]. However, as HCPs begin working together, each may have role expectations about the other that are based on stereotypes, past experiences, and educational backgrounds [38]. As a means to develop trust, useful recommendations should be made consistently over time in order to demonstrate the role and competence of clinical pharmacists [3, 5]. In order for clinical services to be successfully implemented, sustainable and expand, the roles and responsibilities need to be clearly described, and opportunities to support collaborative relationships need to be created. Additionally, decision makers’ support of clinical pharmacy services has been shown to enhance implementation [3, 39].

Factors like gender, age, years of work experience, and previous experience working with a clinical pharmacist were not associated with the understanding of HCPs regarding the role, attitude and competencies of clinical pharmacists. Similar studies found that frequent communication between physicians and clinical pharmacists, as well as exposure to pharmacists participating in clinical activities was a significant factor influencing physicians’ overall attitudes towards clinical pharmacists [13, 40–43]. One potential explanation for this, as well as the study’s overall positive mean scores, could be that this study was conducted in a teaching/academic hospital where HCPs could also have been exposed to the clinical pharmacy profession through contact with clinical pharmacy students, as well as in an academic manner through the university. Another explanation could be that the HCPs have heard of clinical pharmacists and gained some knowledge regarding their role through the clinical pharmacist positions already established in the private healthcare sector [7, 28]. This underscores the growth in the clinical pharmacy profession and marked progress in the acceptance of clinical pharmacists in South Africa over the past decade. This development is thought to have occurred in parallel with the growth of clinical pharmacy graduates (education) in South Africa, as well as the number of hospitals providing clinical pharmacy services [18, 28, 44, 45].

When investigating the differences between departments/units where HCPs work, in this study we found that doctors and nurses working in surgical units’ understanding regarding the role of clinical pharmacists were poorer as compared to clinical pharmacists. Similarly, the perceptions of doctors and nurses in non-surgical units, regarding the role of clinical pharmacists were poorer than both clinical pharmacists, and pharmacists. This indicates that exposure to the role of clinical pharmacists, in both surgical, as well as non-surgical units are not sufficient. Whereas, from an interprofessional collaboration perspective it is found that collaboration with non-surgical departments is often easier [46, 47]. A recent study on clinical pharmacists in South Africa found a lack of time to deliver clinical services as the main barrier to perform their role optimally [7]. Again, highlighting the importance of consistent time spent in the wards, where clinical pharmacists are fully integrated in the hospital environment [48]. Lack of awareness and resources may be barriers to the expansion of clinical pharmacy services, especially in the public healthcare sector of South Africa. Clinical pharmacy service implementation requires the incorporation of a new profession into healthcare provision, representing both a paradigm-shift and a new multidisciplinary team model, where pharmacists participate in clinical decision-making. Similar studies in other countries have identified that when clinical pharmacy services are implemented for the first time, there should be an adoption of new models of care and their respective processes [6, 49–51]. Therefore, progressive interaction is necessary to promote the knowledge of clinical pharmacy services and their benefits. In turn, if the understanding of doctors and nurses and their attitude towards the profession of clinical pharmacy improves, it could generate increased acceptance of clinical pharmacists, as well as their pharmaceutical care interventions contributing to better quality of healthcare [1, 5, 13, 14, 17].

Concerning pharmacists’ and clinical pharmacists’ different levels of agreement on activities that form part of the scope of a clinical pharmacist, some pharmacists were unsure whether certain activities form part of the role of a clinical pharmacist. Highlighting the fact that not all pharmacists have (adequate/equal) knowledge on the role of a clinical pharmacist, which could contribute to differences in their role expectations of clinical pharmacist colleagues and possible conflict within the hospital pharmacy setting. This is underscored by the finding that at least half of the clinical pharmacists disagreed or strongly disagreed that activities pertaining to the profession of a general hospital pharmacist, form part of their role. This is interesting, as these activities were listed from the scope of practice of clinical pharmacists, outlined by the SAPC in 2014 [22]. Additionally, in 2015, the NDoH published the White Paper on NHI, which aims to provide all South Africans with universal health coverage [29]. They also published Quality Standards for Healthcare Establishments [52] where they describe that pharmacists are required to fulfill their role in the domain of patient clinical governance, patient safety and care by reducing medication errors and adverse events. Clinical pharmacists are required to align themselves with the National Core Standards, supporting the aim of the NHI [29], which will require the direct participation of clinical pharmacists in the pharmaceutical care process ensuring continuity of care and better patient outcomes [20, 21].

### Implications for practice and recommendations

For clinical pharmacy to succeed in South Africa, it should be supported by the statutory bodies, professional societies, management structures within the workplace and funding structures [9]. In the absence of standardized clinical activities [7], there is the possibility that among different HCPs, as well as organizational/statutory bodies there might be differences in expectations regarding the role of a clinical pharmacist. This furthermore affects the motivation of clinical pharmacists to deliver clinical services, as a work environment with standardized practice guidelines could enable them to deliver patient care they derive inspiration from [18]. Re-evaluation of the main tasks of clinical pharmacists and general hospital pharmacists might be necessary to clarify roles and manage expectations from both sides. Developing a standard job description could help with setting role expectations and promoting HCPs mutual understanding of their respective roles contributing to forming effective interprofessional collaborations.

Individual characteristics such as educational background could further influence how professionals move towards collaborative working relationships [23, 53]. As previously described, ward-based pharmacists are not yet common in South Africa, and many physicians and nurses are unaware of the capabilities of clinical pharmacists. The majority of pharmacists still work in community pharmacies, with their most common task being; providing advice to the patient, and minimal collaboration with other professionals [23]. Trust and similar perspectives about each other’s roles can be constructed through interaction of the HCPs during training [54, 55]. Recommendations for successful implementation thus include interprofessional practice education opportunities. Interprofessional Education (IPE) has been defined as circumstances “where two or more professions learn with, from and about each other to improve collaboration and the quality of care“ [56]. If clinical pharmacists were to train with other professions, there is opportunity to improve interprofessional collaborative care, as well as understanding around the responsibilities of other professional roles. This would require IPE opportunities within the post-graduate M. Pharm curriculum to engage with e.g. final year students from the Bachelor of Nursing Science (B Cur) and Bachelor of Medicine and Bachelor of Surgery (MBChB) degree programmes. Furthermore, the initiation of staff induction programmes, where a clinical pharmacist is introduced to a ward or another new staff member/HCP, could reduce ambiguity and result in role clarity [23]. This could facilitate the ability of new staff members to comprehend their new environment and develop, or enhance, the role clarification competency needed for effective collaboration with other HCPs [57]. Additionally, to incorporate short, regular interprofessional meetings into the practice level, has been shown to develop and improve collaboration [58]Regular meetings encourages communication and contact which may lead to more flexible interaction between HCPs, managing team expectations [58].

Future research providing an in-depth qualitative look at the influence that the perceived understanding of HCPs on the role of clinical pharmacists’ have on actual clinical service implementation and the motivation of clinical pharmacists is recommended. Further studies should aim to describe the current inclusion of the clinical pharmacist in the multidisciplinary team and perceived barriers to collaborative practice in healthcare institutions across South Africa.

### Strengths and Limitations

Our study took place at a single public healthcare institution, providing a unique setting, which is the second largest public hospital in the country. Other public hospitals in general only employ one or two clinical pharmacy graduates, whereas the study setting has eight. As the specialization of Clinical Pharmacy has not yet been registered, this could have had an effect on the understanding of HCPs regarding the role of clinical pharmacists, but we have not measured this. Although there are no official clinical pharmacist positions currently in place, these eight graduates deliver clinical services to some extent on a daily basis. Even though the number of clinical pharmacists included in the study was small, the sample reflects the demographics of ward-based practice in public institutions in South Africa. It is important to highlight that the sample size further reflects the restraints of the small size of the current service delivery, and the recognized human resource shortages. The number of doctors and nurses that have more than occasional contact with pharmacists is limited. As most studies up till now have focused on the working relationship between community-based pharmacists and physicians, this study fills a gap in the limited existing literature and provides useful information regarding the understanding of HCPs concerning the role of clinical pharmacists. To our knowledge this is the first study exploring this in South Africa. The results of the study may also be relevant in countries where ward-based pharmacists are infrequent, such as in other parts of Africa, Latin America, the Middle East and some parts of Asia and Europe.

## Conclusion

This study was able to provide new insights into the understanding of HCPs regarding the role of clinical pharmacists. The findings highlighted the possible impact of role expectations among HCPs. A standard job description with recognition from statutory bodies could promote other HCPs, as well as clinical pharmacists’ understanding of their roles. The study further identified that interventions like interprofessional education opportunities, staff induction programmes and regular interprofessional meetings might be needed to generate acknowledgement of clinical pharmacy services, promoting the acceptance and growth of the profession.

## Data Availability

The datasets used and analysed during the current study are available from the corresponding author on reasonable request.
